# Risk assessment for condylar stress fracture in elite racing Thoroughbreds using standing computed tomography‐based virtual mechanical testing

**DOI:** 10.1002/evj.70145

**Published:** 2026-01-18

**Authors:** Nicola L. Brown, Soroush Irandoust, Elleana J. Thom, R. Christopher Whitton, Corinne R. Henak, Peter Muir

**Affiliations:** ^1^ Department of Surgical Sciences University of Wisconsin‐Madison Madison Wisconsin USA; ^2^ Department of Mechanical Engineering University of Wisconsin‐Madison Madison Wisconsin USA; ^3^ Department of Veterinary Clinical Sciences, Melbourne Veterinary School, Faculty of Science University of Melbourne Werribee Victoria Australia; ^4^ Department of Biomedical Engineering University of Wisconsin‐Madison Madison Wisconsin USA; ^5^ Department of Orthopedics & Rehabilitation University of Wisconsin‐Madison Madison Wisconsin USA

## Abstract

**Background:**

Condylar stress fracture of the third metacarpal bone (MC3) is a common catastrophic injury in Thoroughbred racehorses and is associated with parasagittal groove (PSG) subchondral osteolysis. Standing computed tomography (sCT) imaging enables sensitive identification of this fatigue‐induced early subchondral bone injury (SBI), but there is no objective method for identifying racehorses at heightened risk of condylar stress fracture.

**Objectives:**

To estimate PSG first principal strain in elite Thoroughbred racehorses that have undergone subjective risk assessment using sCT fetlock screening.

**Study Design:**

Retrospective clinical study.

**Methods:**

We used fetlock sCT images from nine thoracic limbs from seven Thoroughbred racehorses. A tuned, validated, 3D finite element (FE) analysis was used as a virtual mechanical test to estimate PSG first principal strain in the distal MC3 from these joints. Virtual mechanical testing results were compared with a subjective clinical imaging risk assessment using a screening approach by Racing Victoria.

**Results:**

MC3 condyles with PSG SBI consistently and significantly displayed increased levels of first principal strain throughout the PSG. We found focal strain concentrations associated with the SBI location compared to condyles with no evidence of PSG SBI. Diagnosis of SBI with PSG focal osteolysis, FE‐predicted strain elevation, and clinical imaging risk assessment were concordant with *R* ^2^ = 0.62.

**Main Limitations:**

The sample size was small, and our virtual mechanical testing protocol does not account for whole‐joint physiology.

**Conclusions:**

Risk assessment through sCT screening is an established approach to injury prevention in racing Thoroughbreds. Concordance of a current clinical imaging risk assessment approach by Racing Victoria with objective FE analysis of principal strain in sites of PSG SBI in the present study suggests 3D FE analysis using a validated pipeline has potential as a new approach for routine assessment of risk of MC3 condylar stress fracture in Thoroughbred racehorses once computational pipeline automation yields a clinically relevant analysis time.

## 1 INTRODUCTION

Catastrophic musculoskeletal injuries (CMI) are a leading cause of euthanasia in Thoroughbred racehorses, with a pooled incidence of 1.17 CMI per 1000 race starts worldwide from 1990 to 2017.^1^ Condylar stress fracture of the third metacarpal/tarsal bone (MC3/MT3) is a prevalent example of CMI and frequently results in euthanasia.^1^, ^2^ Catastrophic injuries also increase the risk of jockey injury.^3^

Condylar stress fractures of the MC3 occur due to an accumulation of microdamage^4^ that compromises the mechanical integrity of the distal end of the bone, most commonly on the palmar surface,^5^, ^6^ due to the repetitive high loads the bone experiences during galloping.^7^ Subchondral trabecular bone becomes sclerotic in response to the repetitive loading, and under low strain conditions the damaged bone undergoes remodelling to repair the microcracks and restore mechanical integrity, resorbing damaged bone and replacing it with new bone.^8^ When the bone is excessively subjected to high loads, such as the cyclic loads associated with galloping, microdamage accumulates faster than it can be fully repaired through remodelling, resulting in damage accumulation, intense targeted remodelling, and focal resorption. Such changes are commonly observed in the PSG subchondral bone^8^, ^9^ This type of subchondral bone injury (SBI) is visible as focal subchondral bone resorption in the PSG with standing CT (sCT) imaging.^10^

sCT is a highly sensitive method of detecting fatigue‐induced structural changes in subchondral bone. Such changes in the PSG are associated with condylar fracture and local elevated strain when the articular surface is loaded.^5^, ^11^ However, interpretation of the significance of these lesions is subjective and debated among clinicians.^10^ Therefore, an objective method of assessing risk of incipient fracture is needed. CT‐based finite element (FE) analysis has been extensively used to study bone biomechanics and predict and treat orthopaedic complications in the past few decades.^12^, ^13^, ^14^, ^15^, ^16^, ^17^, ^18^, ^19^, ^20^, ^21^, ^22^, ^23^, ^24^, ^25^ More recently, virtual mechanical testing of ex vivo MC3 bones by 3D sCT‐based FE analysis predicted elevated PSG strain in bones with PSG SBI.^26^ Incorporation of different bone material properties associated with sub volumes of sclerotic and lytic bone increased the accuracy of PSG strain prediction. This approach has been validated using ex vivo bone specimens but has not yet been applied to live Thoroughbred racehorses.

We hypothesised that 3D FE analysis of in vivo limbs of actively racing and training elite Thoroughbred racehorses would predict elevated strain in the PSG with presence of SBI, thereby indicating elevated risk of condylar stress fracture. In addition, risk assessment scores made by clinicians were compared with FE‐predicted PSG strain to investigate alignment of the current subjective sCT‐based clinical imaging risk assessment with the objective assessment using virtual mechanical testing.

## 2 MATERIALS AND METHODS

### 2.1 Diagnostic imaging and sample population

Fetlock sCT scans were performed at the University of Melbourne Equine Centre using an Equina® sCT scanner with an exposure of 160 kVp and 8 mA, with 1 mm slice thickness. An electron density phantom (model 062M, CIRS Inc, Arlington, VA, USA) with four plugs of 200, 800, 1250, and 1500 ${\mathrm{mg}}_{\mathrm{HA}}/{\mathrm{cm}}^{3}$ was scanned asynchronously for calibration of CT density ($\rho_{\mathrm{CT}}$, Figures S1 and S2). Nine thoracic limbs from seven elite Thoroughbred horses training and racing in the 2021, 2022, and 2023 Spring Racing Carnival events under the oversight of Racing Victoria were selected as they cover the full range of clinical imaging risk assessment scores (Table 1). The horses ranged in age from 3 years old to 7 years old. There were six geldings and one mare. Digital radiographs were also available for qualitative comparison with sCT for detection of structural changes in the PSG in two horses. Flexed dorsopalmar digital radiographs were made 74 and 43 days before sCT was performed for Horses #2 and #4, respectively.

**TABLE 1:** Clinical imaging risk assessment for Thoroughbreds racing in Melbourne, Australia under the care of Racing Victoria.

ID	Clinical imaging risk assessment scores
1	1 (0, 1, 1)
2^a^	2 (1, 2, 2, 2)
3	1 (0, 1, 1)
4	0 (0, 0, 0)
5	2 (1, 2, 2)
6	2 (1, 2, 2)
7	2 (2, 2, 2)

*Note*: Racing Victoria clinical imaging risk assessment scale was 0—Standard level of injury risk, 1—Moderately elevated level of injury risk, 2—Heightened level of injury risk. Risk assessment was based in review of sCT diagnostic imaging for structural change by a panel of three expert reviewers.

^a^ This horse was reviewed by four experts.

### 2.2 Clinical imaging risk assessment from subject review of sCT images

Assessment of structural changes in the fetlock was undertaken using sCT images by a panel of 3 or 4 expert veterinarian consultants. Expert veterinarian consultants provided assessment of risk of injury for each horse as standard risk (0), intermediate risk (1), and heightened risk (2) (Table 1). Except for PSG lesions, no other particularly concerning fatigue injury was present from the imaging assessment in these horses to contribute to the associated injury risk assessment score.

### 2.3 Virtual mechanical testing

Out of the 14 limbs scanned from the 7 horses (Table 1), due to long model preparation times, only 9 of them were analysed with our virtual mechanical testing pipeline which has been described in detail previously^26^ and is summarised here. The distal MC3 bone was segmented using Mimics (v.26), and based on the density threshold of HU = 1200 ($\rho_{\mathrm{CT}}=1,055.75\;{\mathrm{mg}}_{\mathrm{HA}}/{\mathrm{cm}}^{3}$), the sclerotic subchondral trabecular bone in the palmar aspect (HU ≥1200), and the lytic regions in the PSG (HU ≤1200, if present) were isolated. If an isolated lytic region in the PSG is detected using this threshold, the condyle was referred to as a CASE, otherwise as a CTRL. This threshold was different from what was used in the previous study to optimally capture the sclerotic subchondral bone. Hence, sensitivity of the predicted PSG strain to this threshold was assessed. We made additional models by (1) segmenting the sclerotic volume in one CTRL condyle by using HU = 1100 and HU = 1300 values, and (2) segmenting the lytic volume in one CASE condyle by using HU = 1150 and HU = 1250 values. It is important to note that sCT scans acquired at different times would be expected to have different calibration equations resulting in variation in HU values for matched bone mineral density values, however that variation is less than 45 HU,^27^ thus the results of the sensitivity study are informative for the error expected from scanning at different times.

**FIGURE 1:**
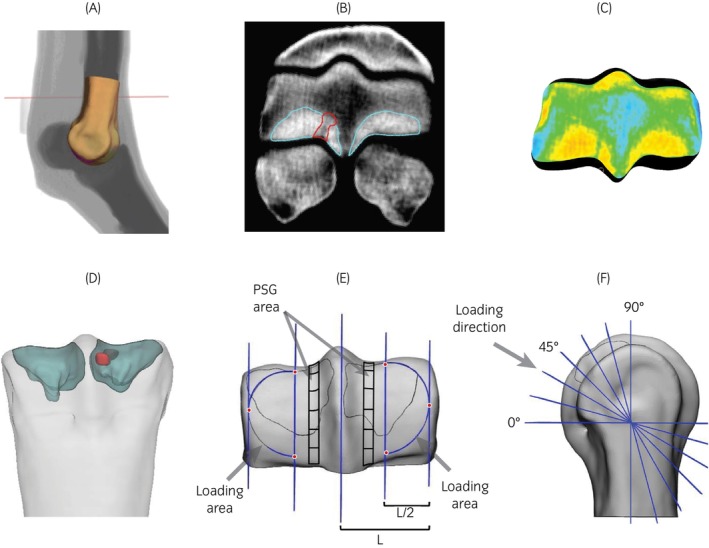
3D segmentation of the distal third metacarpal bone from the standing CT image set (A) and subsequent segmentation of the subchondral sclerotic bone (cyan) and parasagittal groove (PSG) lysis (red), if present (B). The ratio of the volume of each sclerotic region to the volume of its corresponding condyle was used to calculate the adaptive factor. An adaptive factor was applied to the regions of sclerosis. CT density was mapped in the model at each element individually (C). In the 3D model (the dorsopalmar view is shown in panel (D) 3 mm wide strips centred at the PSG of both condyles were defined and divided into six equally angled regions in 15 degrees intervals from the most palmar end to the transverse ridge (E, F). The loading area was created with a straight edge in the dorso‐palmar direction at the half distance between the sagittal ridge and the most lateral/medial aspect (L), and a curve fit on the three points shown on the image.

Three dimensional distal MC3 and the sclerotic and lytic volumes were then smoothed and imported in 3‐matic (v.18), and a uniform triangular surface mesh with 0.25 mm edge length was created for all surfaces (mesh density based on prior mesh convergence analysis^26^). Anatomical planes were established by first creating the transverse plane by finding its normal vector as the axis of a cylinder fit to the distal MC3. The sagittal plane was created normal to the transverse plane, and through two points on the palmar and dorsal aspects of the sagittal ridge. The frontal plane was then defined perpendicular to the transverse and sagittal planes. The distal 2.5 in (6.35 cm) of the MC3, measured from the most distal part of the sagittal ridge along the long axis of the bone, was separated for virtual mechanical testing. For each condyle, a 3 mm strip in the dorsopalmar direction centred around the PSG on the palmar aspect of the condyle, covering the 90 degrees between a frontal plane through the transverse ridge and a transverse plane through the most palmar end, was marked to monitor PSG strain. These 3 mm strips were split into six equally angled regions of 15 degrees each for regional strain analysis (Figure 1). Additionally, the area between the transverse ridge and the most palmar aspect in the distopalmar direction, and half of the length between the most lateral/medial aspect and the sagittal ridge in the lateromedial direction was marked for loading (Figure 1).

The 3D distal MC3s were imported in FEBio Studio (v.2.6) and discretised into 0.25 mm linear tetrahedral elements for the sclerotic and lytic regions, and 1 mm linear tetrahedral elements for the remainder of the distal MC3 bone. Elementwise (heterogeneous) isotropic linear elastic material properties were assigned with Young's modulus tuning in the sclerotic and lytic regions according to our validated methodology.^26^ This requires an adaptive factor defined for the sclerotic region (−1.00 < *A*_*SCL*_ <0.50), and a damage factor for the lytic region (*D*_*LYS*_ = 0.65). Element‐wise ash density ($\rho_{\mathrm{ash}}$) was calculated from CT density ($\rho_{\mathrm{CT}}$), and Young's modulus was calculated for the sclerotic regions, PSG SBI regions (if present), and the rest of the distal MC3 individually. A constant Poisson's ratio of 0.3 was used.

The proximal surface of the model was constrained and a uniformly distributed 7.5 kN load with convergence required at 2.5 kN increments was applied to the previously marked area for loading in each condyle at 60 and 30 degrees with respect to the frontal and transverse planes respectively, replicating prior ex vivo mechanical testing loading conditions.^5^ FE‐predicted strain in 6 regions of the PSG was examined for each condyle (Figure 1) and strain levels were compared between CASE and CTRL condyles.

### 2.4 Statistical analysis and risk classification

Mean PSG first principal strain was compared between CASE and CTRL condyles using the Wilcoxon Rank‐Sum test. The first principal strain is the maximum strain at a given point and a predictor of bone fracture.^28^ Next, a logistic regression model was built as a classifier of condyles with heightened level of injury risk to find the association between clinical imaging risk assessments and the associated FE‐predicted PSG strain. Due to the small sample size, risk categories of 0 and 1 were merged. In horses assigned a risk score of 2, only the condyles with PSG lesions (determined by the modelling pipelines as explained in Section 2.3) were assumed to be the condyles at heightened injury risk. The R‐squared of the logistic regression model was measured to find the concordance between the two assessments. The power of the classifier was calculated with a significance threshold of <0.05 and resampling strain for 1000 simulations. The effect of the sample size on the classifier power was estimated with resampling strain with sample sizes at *n* = 5 intervals ranging from *n* = 10 to *n* = 50. Analysis was done with MATLAB R2023b (Mathworks, Natick, MA, USA).

## 3 RESULTS

**FIGURE 2:**
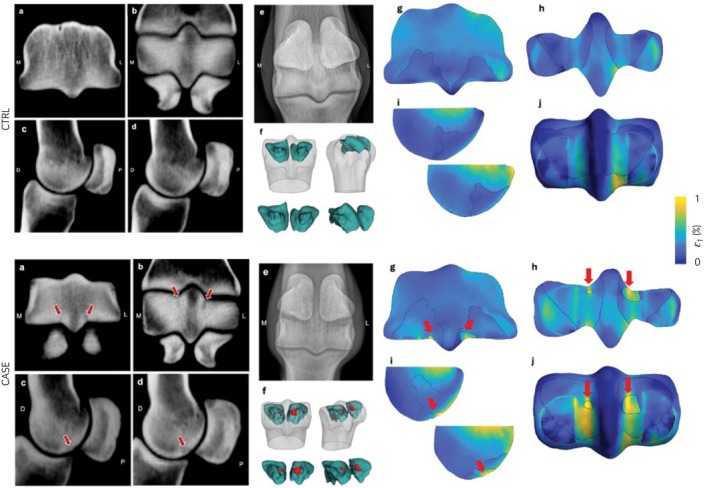
sCT images of the left metacarpophalangeal joint for a horse with control (CTRL) condyles (Horse #4 LF) and a horse with biaxial parasagittal groove (PSG) subchondral bone injury (SBI) (CASE, Horse #2 LF. Frontal oblique (A), transverse oblique (B) and two parasagittal views at the lateral and medial PSGs (C, D) from the left metacarpophalangeal joint sCT images, are shown. The flexed dorsopalmar digital radiograph (E) of each limb is shown for comparison with the sCT slices. No PSG osteolysis is evident on the radiographs. The 3D geometry of the FE model with subchondral lytic, if present, and sclerotic volumes (F). The FE‐predicted first principal strain is shown in the same sCT slices (G, H, and I) and on the joint surface (J). Red arrows indicate PSG SBI and the associated elevated strain. L, M, D, and P labels on the sCT images indicate lateral, medial, dorsal, and palmar, respectively. These horses were not affected with palmar osteochondral disease (POD).

Seven of the 18 condyles (from 9 limbs) studied were determined to have PSG SBI (CASE) by the segmentation step of the virtual mechanical testing pipeline (Table 2). Condyles with PSG SBI showed elevated first principal strain in the PSG and focally around the SBI lesion compared to CTRL condyles (Figure 2). The PSG SBI and the associated focal elevation of strain were most obvious in the transverse oblique slice in both the sCT images and the 3D FE model. Qualitative comparison of the FE‐predicted strain distribution on the joint surface also showed elevated PSG strain in the CASE condyles compared with the CTRL condyles. In addition to the palmar aspect of the PSG of the CASE condyles, strain was elevated in the dorsal aspect in the grooves, evident in the transverse oblique view. With respect to diagnostic imaging, the flexed dorsopalmar digital radiographs did not reveal evidence of PSG SBI and subchondral osteolysis in the horse with SBI. The sCT image sets and FE‐predicted strains plots for the rest of the limbs are shown in Figures S3–S9.

**TABLE 2:** Presence (CASE) or absence (CTRL) of parasagittal groove (PSG) subchondral bone injury (SBI) in the condyles of the limbs studied in this paper, determined through the segmentation step of the virtual mechanical testing pipeline.

ID	Limb	LC PSG	MC PSG
1	LF	CTRL	CTRL
2	LF	CASE	CASE
	RF	CASE	CTRL
3	RF	CTRL	CTRL
4	LF	CTRL	CTRL
5	LF	CTRL	CASE
	RF	CASE	CTRL
6	RF	CASE	CTRL
7	LF	CASE	CTRL

Abbreviations: LC, lateral condyle; LF, left thoracic limb; MC, medial condyle; PSG, parasagittal groove; RF, right thoracic limb.

**FIGURE 3:**
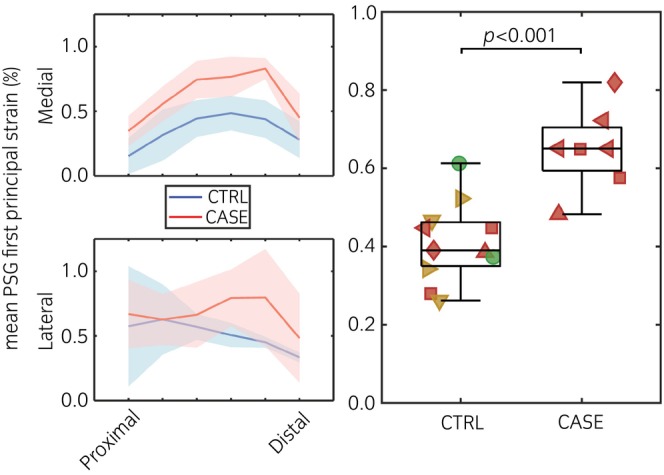
Mean PSG first principal strain was qualitatively higher in both medial and lateral sides of the CASE condyles. Strain peaked closer to the transverse ridge in the medial side in both CASE and CTRL condyles, and in the lateral side of the CASE condyles. The shaded area indicates the 95% confidence interval. In the box plot, condyles (two or four) of the same horse have the same shape, and the green, yellow, and red colours indicate standard (0), moderate (1), and heightened (2) risk of injury, respectively, based on the median clinical imaging assessment scores shown in Table 1. Mean PSG strain was higher in the CASE condyles (*p* < 0.001), and all horses with CASE condyles were assessed to have heightened injury risk. All the red datapoints in the CTRL group had a contraaxial CASE condyle.

First principal strain was increased in the CASE condyles compared with the CTRL condyles (*p* < 0.001, Figure 3). In the medial PSGs, first principal strain peaked close to the transverse ridge in both CASE and CTRL condyles. In the lateral PSGs, strain was lower close to the transverse ridge and increased towards the most palmar/proximal end in the CTRL condyles, while it peaked around the lesion in the CASE condyles. The threshold values used for segmentation of the sclerotic and lytic volumes had a small influence on the predicted PSG strain (Figure S10). All horses with CASE condyle(s) were assessed to have heightened risk of injury from condylar stress fracture from subjective assessment of screening sCT imaging.

The logistic regression model had a power of 0.4 and *R* ^2^ = 0.62, and its power is estimated to reach 0.80 at a sample size of *n* = 30 (Figure S11).

## 4 DISCUSSION

In this report, we evaluated use of a virtual mechanical testing approach that has been validated against experimental data.^5^, ^26^ The fetlock joints of the horses we studied with PSG SBI had lesions that are typical of affected horses.^4^, ^29^, ^30^ As previously described,^10^ Horse #2 with PSG SBI in the present study was readily identified by sCT, but not by digital radiography using the flexed dorsopalmar view. This aligns with past work where reliance on fetlock digital radiography likely underestimates risk of serious injury from condylar stress fracture in racing Thoroughbreds.^10^

The results of this study support our hypothesis that FE‐predicted PSG strain was larger in condyles with PSG SBI compared to those without. The elevated strain is indicative of mechanical compromise associated with SBI in the PSG,^5^, ^30^ and may be indicative of elevated risk of condylar stress fracture, as strain levels exceeding the bone's capability is the cause of the condylar stress fracture. These observations are also supported by the reduction in catastrophic injuries that has occurred since routine sCT screening and subjective review of sCT structural changes was used for clinical imaging risk assessment by Racing Victoria in 2021 until now.^31^

Condylar stress fracture risk has been linked to subchondral bone functional adaptation by bone modelling.^32^, ^33^ Subchondral sclerosis acts to increase the bone volume fraction of the subchondral bone to better resist the high cyclic loads associated with racing and we found it necessary to consider this feature specifically in our virtual mechanical testing pipeline to properly estimate first principal strain in the MC3 bone end.^26^ Similarly, the dimensions of the PSG subchondral focal osteolysis lesion are thought to cause a reduction in MC3 stiffness^30^ and we found that consideration of the subchondral osteolytic volume was important to our virtual mechanical testing pipeline.^26^ Contralateral PSG subchondral lucencies and associated fatigue injury are known to be associated with catastrophically injured horses,^32^ and we found that the bones of horses with this lesion had consistently elevated first principal strain in the affected PSG.

In the horses of this report, we identified good concordance between the 3D FE virtual mechanical testing and the subjective clinical imaging risk assessment through the Racing Victoria's Injury Prevention Program. This is an important finding as past work suggests clinician observers are generally better at detecting and assessing structural changes from fetlock diagnostic imaging than providing a rating for risk of imminent injury.^10^ Fetlock MC3 SBI was most easily visible on the transverse oblique slice of sCT images, supporting the use of this view as a standard screening tool as in past work.^10^, ^34^

The good concordance between the FE‐predicted strain and the clinical imaging risk assessments suggests that FE analysis for virtual mechanical testing is clinically useful for identification of horses with heightened injury risk. Both false positive and false negative identification of horses with heightened injury risk are potentially concerning. Allowing horses with heightened injury risk to continue to race will lead to ongoing serious or fatal injury and concerns regarding Thoroughbred and jockey welfare. Alternatively, removing horses from racing unnecessarily is financially concerning. In the horses of the present study, one CTRL condyle had FE‐predicted PSG strain that approximated the CASE group, and one CASE condyle had FE‐predicted PSG strain that approximated the CTRL group. This suggests more data are needed to accurately quantify the stress fracture risk based on the predicted PSG strain for personalised care.

While initial clinical outcomes from the risk assessment approach developed from the expertise of Racing Victoria and the consultants that make up their imaging review panel are promising, longitudinal clinical imaging risk assessments are needed to fully validate the approach as a clinically useful and accurate classifier. One of the specific challenges in further in vivo longitudinal clinical studies is that allowing horses assessed as having a high risk of imminent serious injury to continue to participate in racing and monitoring these animals to see if a stress fracture develops is not ethical and would not be viewed favourably by society. Use of virtual mechanical testing assessment of sCT images has great potential to address such a challenge by monitoring subtle changes in the mechanical function of the distal MC3 over time that may not be obvious to clinicians. Another advantage of the virtual mechanical testing pipeline is that it provides an objective screening tool that does not require the opinion of experts that may not be available when needed.

In the horses of the present study, we identified qualitative differences in the pattern of first principal strain between the lateral and medial PSGs. In the lateral PSGs, strain increased from the transverse ridge distally towards the most palmar/proximal end in the CTRL condyles while it peaked around the lesion in the CASE condyles, whereas in the medial PSGs strain peaked close to the transverse ridge in both CASE and CTRL condyles. The presence of some degree of PSG strain elevation in all lateral condyles, as compared to medial condyles, may suggest a mechanism by which SBI preferentially develops in the PSG of the lateral condyle. This fits with fracture epidemiology knowledge, as the incidence of lateral condylar stress fracture is higher than that of medial condylar stress fracture.^35^

There were several limitations to this study. In the two horses with digital radiographs, the radiographic images were not made at the same time as the sCT images. Our analysis was based on a relatively small sample of elite Thoroughbred racehorses that may not cover all potential clinical features or range of lesion severities. Atypical condylar stress fractures can be associated with palmar/plantar osteochondral disease (POD) lesions. Virtual mechanical testing of bones with POD lesions was not specifically investigated in this study. It is possible that analysis of a larger sample size of Thoroughbreds may identify racehorses with discordant findings between clinical imaging risk assessment and 3D FE analysis. Computational pipeline automation will readily enable this research goal. Discovery of discordant cases would likely inform development of a comprehensive condylar stress fracture risk assessment approach and provide more data on the accuracy of virtual mechanical testing as a screening tool for risk assessment. An additional challenge stems from assigning region‐wide damage and adaptive factors to sclerotic and lytic regions, which do not account for the gradient of damage which is likely more severe closer to the articular surface. Another limitation on the material modelling is that the post‐yield behaviour of the bone is not included and models, therefore, cannot predict fracture load, although this does not preclude its clinical utility as a CASE/CTRL classifier. Future studies that analyse variation in fatigue damage within subchondral sclerotic and lytic regions, including horses that have developed POD with and without ipsilateral PSG subchondral osteolysis as well as 3D FE models that integrate physiological stress fracture, are needed to better encompass accurate material properties of sclerotic and lytic regions of bone and better determine whether risk of fracture is truly imminent. The virtual mechanical testing we describe does not account for whole‐joint physiologic loading, which should also be explored in future work. Incorporation of the proximal sesamoid bones and the proximal phalanx as well as tendons, ligaments and muscles would create a model that more closely reflects true in vivo physiologic loading. Lastly, future studies should target automation of the pipeline presented to make it a clinically relevant diagnostic tool since fast patient‐specific fracture risk assessments would be needed to assess imminent risk of injury before a race. Computing resources are becoming better and more widespread, making the computational time a diminishing concern.

## 5 CONCLUSION

The sCT‐based virtual mechanical testing approach described in this report provides an objective, non‐invasive method of assessing imminent risk of condylar stress fracture in Thoroughbred racehorses and aligns well with the risk assessment grading system developed by Racing Victoria that uses subjective assessment of sCT structural change. Continued application of this approach to more bones will provide insight into interpretation of structural changes visible with sCT imaging, determining whether the shape of SBI or relative volumes of SBI and sclerosis have an impact on strain prediction and therefore fracture risk. Mandatory sCT screening and risk assessment before racing is a clinically feasible approach that promises accurate identification of horses with high imminent risk of bone fracture and enables removal of high‐risk horses from racing for personalised care, ultimately reducing the incidence of condylar stress fracture, euthanasia in Thoroughbred racehorses, and injury of jockeys.^3^ With future pipeline automation to provide clinically relevant reporting times, virtual mechanical testing has the potential to make important contributions to such risk assessments.

## FUNDING INFORMATION

This work was funded by The Hong Kong Jockey Club Equine Welfare Research Foundation (MRG‐2022‐100008) and the AVMA/AVMF 2nd Opportunity Research Fund.

## CONFLICT OF INTEREST STATEMENT

Peter Muir is a Founder of Asto CT, a subsidiary of Centaur Health Holdings Inc. and a founder of Eclipse Consulting LLC.

## AUTHOR CONTRIBUTIONS

**Nicola L. Brown:** Methodology; investigation; visualization; writing – review and editing; writing – original draft. **Soroush Irandoust:** Conceptualization; methodology; validation; formal analysis; investigation; supervision; visualization; writing – review and editing; writing – original draft. **Elleana J. Thom:** Visualization. **R. Christopher Whitton:** Conceptualization; resources; writing – review and editing. **Corinne R. Henak:** Conceptualization; methodology; formal analysis; resources; writing – original draft; writing – review and editing; supervision; funding acquisition. **Peter Muir:** Conceptualization; methodology; formal analysis; resources; writing – original draft; writing – review and editing; project administration; supervision; funding acquisition.

## DATA INTEGRITY STATEMENT

Peter Muir and Soroush Irandoust had full access to all data in the study and take responsibility for the integrity of the data and accuracy of the data analysis.

## ETHICAL ANIMAL RESEARCH

Research ethics committee oversight not required by this journal: retrospective study of clinical records.

## INFORMED CONSENT

Explicit owner consent for inclusion of animals in this study was not stated. All owners/trainers/agents were made aware that case information may be used anonymously for the purposes of clinical research.

## Supporting information


**FIGURE S1.** Regions isolated using local thresholding. The blue arrows indicate the regions of bone with HU ≥1200, which was qualitatively determined to encompass sclerotic bone. The red arrows indicate the region of bone with HU ≤1200 within the parasagittal groove, which was qualitatively determined to encompass damaged bone associated with parasagittal groove subchondral bone injury.

**FIGURE S2.** Calibration of the CT number (HU) to phantom plug density.

**Figures S3 to S9.** show sCT images and the FE‐predicted strain plots for Horses #1–3, #5, #6, #7. Frontal oblique (a), sagittal (b), and transverse oblique (c) views of the sCT images are shown. The 3D model of the distal MC3 bone was created with the regions of high sclerosis and parasagittal groove lysis (if present) separated (d). First principal strain is shown in the frontal oblique (e), sagittal (f), and transverse oblique (g) slices, as well as the palmar joint surface (h). Flexed dorsopalmar digital radiograph of the fetlock, if available (i) LF—left thoracic limb, RF—right thoracic limb. *Note*: Image montages from Horse #2 LF and #4 LF are presented in Figure 2 in the manuscript. Condyles with palmar osteochondral disease (POD) are also indicated.

**FIGURE S10.** Sensitivity of FE‐predicted mean PSG first principal strain in the medial (left, CTRL) and lateral (right, CASE) condyles of Horse #7 LF to the three different thresholds used for segmentation of the sclerotic (left, CTRL) and lytic regions.

**FIGURE S11.** The classifier's power is expected to reach 0.8 at a sample size of *n* = 30.

## Data Availability

Some of the data that support the findings of this study are available from Racing Victoria. Restrictions apply to the availability of these data, which were used under licence for this study. The clinical data that support the findings of this study are available upon reasonable request from the corresponding author. Open data sharing exemption granted by the editor.
