# Supplementary material for: Risk assessment for condylar stress fracture in elite racing Thoroughbreds using standing computed tomography‐based virtual mechanical testing
Source: Equine Vet J. 2026 Jan 18;58(3):674–81. doi: 10.1002/evj.70145 (PMC13041603; doi:10.1002/evj.70145)

Equine Veterinary Journal

Figure S1. Regions isolated using local thresholding. The blue arrows indicate the regions of bone with HU ≥ 1,200, which was qualitatively determined to encompass sclerotic bone. The red arrows indicate the region of bone with HU ≤ 1,200 within the parasagittal groove, which was qualitatively determined to encompass damaged bone associated with parasagittal groove subchondral bone injury.

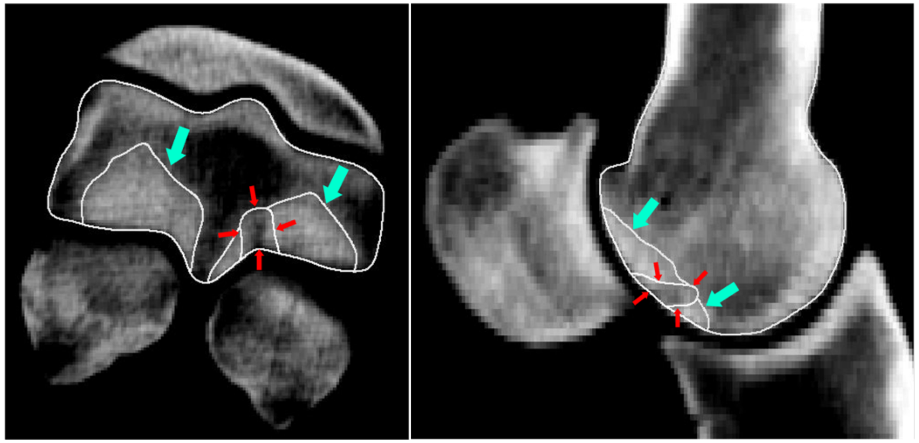

Supplement: Supplementary file 1 — FIGURE S1. Regions isolated using local thresholding. The blue arrows indicate the regions of bone with HU ≥1200, which was qualitatively determined to encompass sclerotic bone. The red arrows indicate the region of bone with HU ≤1200 within the parasagittal groove, which was qualitatively determined to encompass damaged bone associated with parasagittal groove subchondral bone injury. [file EVJ-58-674-s003.pdf]
