# Supplementary figures and images for: Risk assessment for condylar stress fracture in elite racing Thoroughbreds using standing computed tomography‐based virtual mechanical testing
Source: Equine Vet J. 2026 Jan 18;58(3):674–81. doi: 10.1002/evj.70145 (PMC13041603; doi:10.1002/evj.70145)

**Figure S2.** Calibration of the CT number (HU) to phantom plug density.

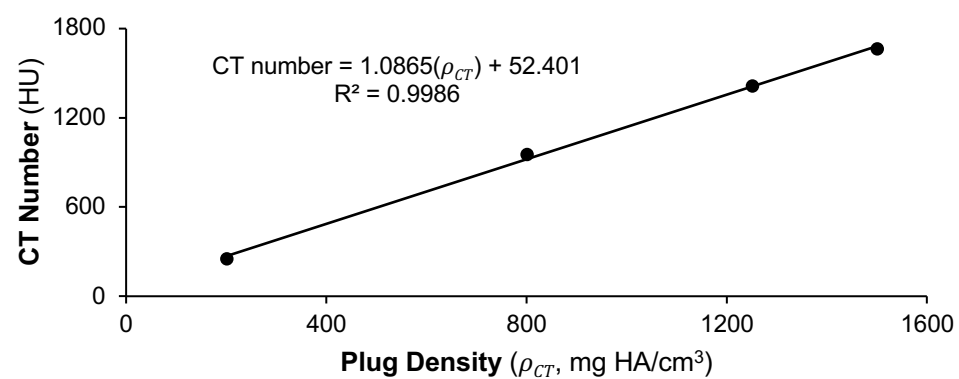

$$\rho_{CT} = (CT \text{ number} - 52.4010)/1.0865$$

(equation S1)

Supplement: Supplementary file 2 — FIGURE S2. Calibration of the CT number (HU) to phantom plug density. [file EVJ-58-674-s004.pdf]

**Figure S11.** The classifier's power is expected to reach 0.8 at a sample size of  $n=30$ .

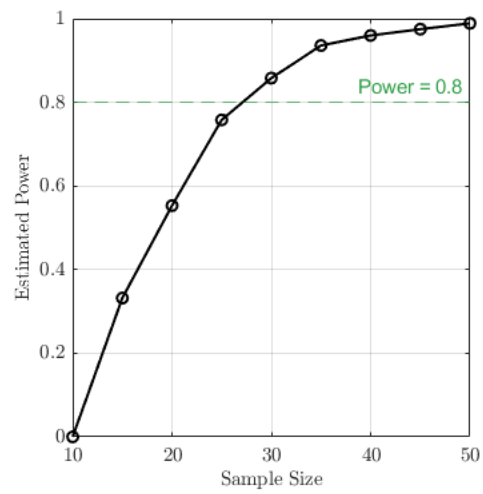

Supplement: Supplementary file 5 — FIGURE S11. The classifier's power is expected to reach 0.8 at a sample size of n = 30. [file EVJ-58-674-s002.pdf]
