# Supplementary material for: Risk assessment for condylar stress fracture in elite racing Thoroughbreds using standing computed tomography‐based virtual mechanical testing
Source: Equine Vet J. 2026 Jan 18;58(3):674–81. doi: 10.1002/evj.70145 (PMC13041603; doi:10.1002/evj.70145)

Equine Veterinary Journal

BEVA

Figure S10. Sensitivity of FE-predicted mean PSG first principal strain in the medial (left, CTRL) and lateral (right, CASE) condyles of Horse #7 LF to the three different thresholds used for segmentation of the sclerotic (left, CTRL) and lytic regions.

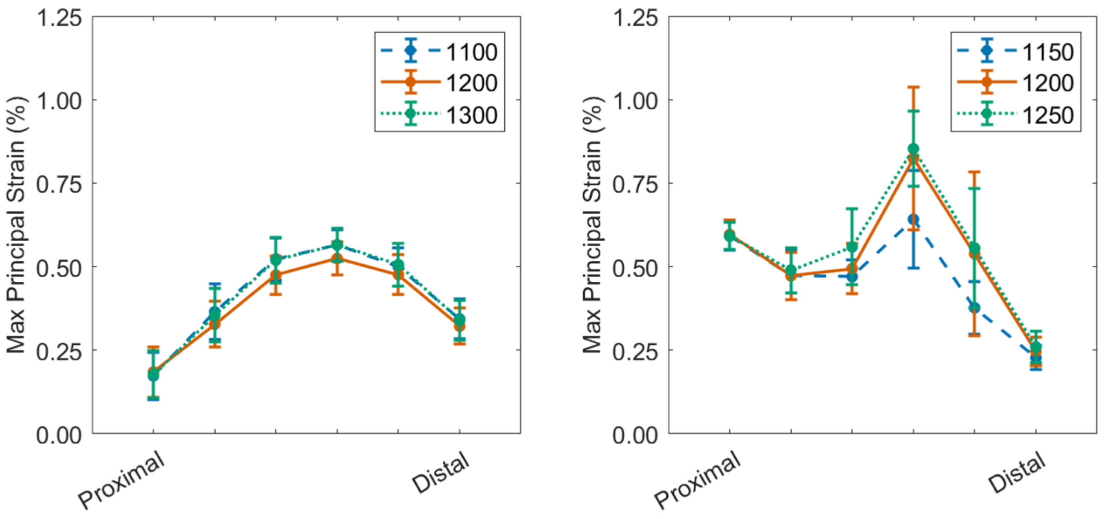

Supplement: Supplementary file 4 — FIGURE S10. Sensitivity of FE‐predicted mean PSG first principal strain in the medial (left, CTRL) and lateral (right, CASE) condyles of Horse #7 LF to the three different thresholds used for segmentation of the sclerotic (left, CTRL) and lytic regions. [file EVJ-58-674-s005.pdf]
